# Progestin Pollution in Surface Waters of a Major Southwestern European Estuary: The Douro River Estuary (Iberian Peninsula)

**DOI:** 10.3390/toxics13030225

**Published:** 2025-03-19

**Authors:** Frederico Silva, Rodrigo F. Alves, Eduardo Rocha, Maria João Rocha

**Affiliations:** 1 Department of Microscopy, School of Medicine and Biomedical Sciences (ICBAS), University of Porto (U. Porto), 4050-313 Porto, Portugal; fredericonunessilva@gmail.com (F.S.); rodrigo.r.f.alves@hotmail.com (R.F.A.); erocha@icbas.up.pt (E.R.); 2 Group of Animal Morphology and Toxicology, Interdisciplinary Centre for Marine and Environmental Research (CIIMAR/CIMAR), University of Porto (U. Porto), 4450-208 Porto, Portugal

**Keywords:** aquatic toxicology, gonanes, progestogen, progesterone derivatives, risk quotients (*RQs*), spirolactone derivatives

## Abstract

The concentrations and spreading of eight synthetic and two natural progestins (PGs) were investigated in surface waters from ten sites at the Douro River Estuary. Samples were filtrated and subjected to solid-phase extraction (SPE) to isolate and concentrate the target PGs. The extracts were cleaned by silica cartridges and analyzed by LC-MS/MS. The finding of biologically relevant amounts of gonanes (22.3 ± 2.7 ng/L), progesterone derivatives (12.2 ± 0.5 ng/L), drospirenone (4.1 ± 0.8 ng/L), and natural PGs (9.4 ± 0.9 ng/L) support the possibility of these compounds acting as endocrine disruptors. Despite the absence of significant differences amongst sampling sites and seasons, the principal component analysis (PCA) and the linear discriminant analysis (LDA) approaches reveal that spring and summer have different patterns of PG distribution compared to autumn and winter. The assessment of risk coefficients (*RQs*) and the potential concentrations of synthetic progestins in fish blood sustains that all tested compounds pose a significant risk to local biota (*RQs* > 1). Additionally, three progestins—norethindrone, norethindrone acetate, and medroxyprogesterone acetate—should reach human-equivalent therapeutic levels in fish plasma. Overall, the current data show PGs’ presence and potential impacts in one of the most important estuaries of the Iberian Peninsula.

## 1. Introduction

Coastal ecosystems are facing growing threats from various stressors [1], with the pollution of natural and synthetic progestins (PGs) being an increasingly significant concern as they often go undetected as environmental micropollutants, needing sensitive methods for their detection and quantification [2,3]. Synthetic PGs are predominantly used for contraception in humans and animals and for various medical applications. Their prevalence is on the rise, driven by growing global demand, as evidenced by projections from the “Progesterone Market”, which forecasts an increase of 8.92% from 2025 to 2030 [4].

Upon entering the aquatic environment, PGs can disrupt endocrine systems in both humans and animals [2]. In humans, exposure to PGs through contaminated fish could hypothetically lead to health issues, and in aquatic animals, PGs affect reproduction, alter sex ratios, and reduce fertility, potentially harming populations and ecosystems [4,5]. Long-term exposure to progestins in water poses serious ecological and public health risks [2]. Consequently, the National Institute of Environmental Health Sciences classifies PGs as endocrine disruptor compounds (EDCs) [6]. Research supports that these micropollutants can cause endocrine disruptions in fish at levels as minimal as 0.8–1.0 ng/L, as they are designed to operate effectively at very low doses, specifically targeting cellular receptors [2,7,8,9]. Moreover, in some aquatic environments, the plasmatic concentrations of these EDCs can be (mainly in fish) equivalent to those that promote therapeutic effects in humans [2]. The last aspect is very worrying due to the risks of having high PGs activity in non-targeted species. For these reasons, there is a continuous challenge faced by wastewater treatment plants (WWTPs) in removing PGs of influents coming from densely inhabited urban centers [2,7].

The Douro River has the largest watershed on the Iberian Peninsula, which drains from many urban and agricultural areas and has a high potential for these micropollutants. This international river flows through Spain, which accounts for 80% of its length, and Portugal, which makes up the remaining 20%. It stretches for 897 km (557 miles) from north-central Spain to Oporto in Portugal, where it flows into the Atlantic Ocean. In its final 22 km, the estuary is flanked by the cities of Oporto and Gaia, which have several wastewater treatment plants and support a population of over 500,000 people [10] and countless domestic animals. There is no published information regarding PGs’ occurrence in the Douro River Estuary. In contrast, other prominent EDCs, including natural and synthetic estrogens such as estradiol, estrone, and ethinylestradiol, were recently measured in this area and in risky amounts for the biota [11].

The primary goals of this study are to expand the range of EDCs assessed in the Douro River Estuary and to provide the first-ever investigation on the water concentrations of four gonanes (testosterone derivatives), namely gestodene (GES), levonorgestrel (LNG), norethindrone (NTD), and norethindrone acetate (NTDA), three progesterone derivatives, which include medroxyprogesterone (MEP), medroxyprogesterone acetate (MPA), and megestrol acetate (MGA), one spirolactone derivative, drospirenone (DSP), and two natural progesterones, specifically 17α-hydroxyprogesterone (17-OHP) and 17α,20β-dihydroxy progesterone (17,20-diOHP). Our objectives also encompass examining the spatial and seasonal distribution of these compounds across a significant area of the Douro River Estuary over one year. Lastly, we aim to assess the potential ecological and biological risks these compounds pose to local aquatic organisms. Accordingly, the environmental risk quotients (*RQs*) predicted to affect concentrations of synthetic PGs in fish plasma were calculated using mathematical procedures.

## 2. Materials and Methods

### 2.1. Materials and Chemicals

All chemicals used were of analytical grade. Hexane (Hex) was supplied by Sigma-Aldrich (Steinheim, Germany). Acetonitrile (AcN), ethyl acetate (EAc), and methanol (MeOH) were obtained from Romil Ltd. (Cambridge, UK). Ultrapure water (conductivity = 0.054 μS/cm, at 25 °C) was produced using a Milli-Q system. The Oasis HLB (Hydrophilic-Lipophilic Balance) solid-phase extraction (SPE) cartridges (6 mL, 200 mg) and the silica cartridges (3 mL, 500 mg) were sourced by Waters Corporation (Milford, MA, USA).

Sigma-Aldrich (Steinheim, Germany) supplied the eight synthetic PGs (DSP, GES, LNG, MEP, MGA, MPA, NTD, and NTDA), the two natural progestins (17-OHP, 17,20-diOHP), and the internal standard (IS), progesterone-d_9_ (P-d_9_).

### 2.2. Sample Collection

**Figure 1:**
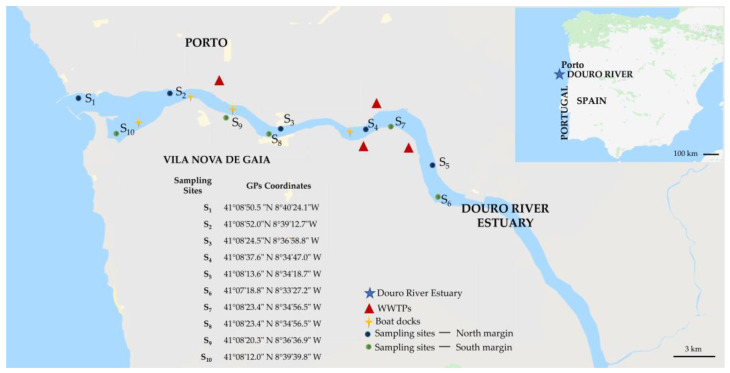
Location of the sampling sites along both margins of the Douro River Estuary (S1 to S10), Portugal. Salinity levels in the studied areas varied from 1.8 ± 2.2 PSU in the inner estuary to 10.0 ± 4.1 PSU near the coastline. This image also points out anthropogenic sources that could contribute to increased concentrations of PGs (adapted from Google KML Map).

Surface water samples (2 L) were taken in independent duplicates at each sampling site as grab samples during low tide from ten sites of the Douro River Estuary (Figure 1). The replicates were collected in winter (February), spring (April), summer (July), and autumn (November) 2023. The duplicates were stored in 2.5 L amber glass bottles which had been pre-rinsed in the laboratory with ultrapure water and further rinsed on-site with the collected water. These waters were maintained at ca. 4 °C during transport and extracted on the same day after being filtered with GF/C™ Glass Microfiber Filters (1.2 μm, Whatman). Prior to analysis, all samples were maintained at −70 °C.

#### 2.2.1. Sampling Areas at the North Margin (Porto City) of the Douro River Estuary

S1 is situated near the entrance of the Douro River into the Atlantic Ocean.

S2 is located at the bird observatory of the Douro River Estuary. This area is close to the effluent from the Sobreiras WWTP, which processes urban sewage from approximately 200,000 inhabitants https://www.aguasdoporto.pt/etar/etar-sobreiras (accessed on 16 March 2025).

S3 is approximately halfway between the previously mentioned Sobreiras WWTP and the Freixo WWTP located at S4.

S4 is situated near the discharge point of the Freixo WWTP effluent into the Douro River Estuary. This plant was designed to handle urban sewage from approximately 170,000 inhabitants “https://aguasdoporto.pt/etar/etar-freixo-porto (accessed on 16 March 2025)”.

S5 is in the estuary’s inner part, ca. 15 km from the Atlantic Ocean. This area is mainly agricultural, with several animal farms.

#### 2.2.2. Sampling Areas at the South Margin (Gaia City) of the Douro River Estuary

S6 is a fluvial beach (Areínho de Avintes) with no apparent sources of urban pollution. Like S5, there are some agricultural activities and animal farms.

S7 is a fluvial beach close to the entrance of the effluent of the Febros WWTP “https://www.simdouro.pt/dados.php?ref=etarfebros (accessed on 16 March 2025)”. This WWTP processes the urban waste of ca. 80,000 inhabitants.

S8 is located south of the discharge point of the Areínho WWTP “https://www.simdouro.pt/dados.php?ref=etarareinho (accessed on 16 March 2025)”. This WWTP handles the sewage of ca. 31,000 inhabitants.

S9 is an important tourist destination, featuring numerous small boats and several docking areas.

S10 is near a protected natural reserve, which has limited access and shows no apparent signs of human pollution.

### 2.3. Sample Preparation (Extraction and Cleanup)

All target PGs were extracted simultaneously using Oasis HLB (6 mL, 200 mg) cartridges connected to an offline SPE vacuum extraction system (Waters), following the procedure developed by [12]. Initially, each cartridge was preconditioned with 6 mL of EAc, followed by 6 mL of AcN and 12 mL of ultrapure water. Then, PGs in surface water samples (2 L), which had been spiked with P-d_9_, were loaded through the last cartridges at 5–10 mL/min flow rate and rinsed with 10 mL of ultrapure Milli-Q water and dried under a nitrogen (N_2_) flow for 30 min. PGs were eluted using 15 mL of EAc. The resulting extracts were then dried and reconstituted in 0.2 mL of EAc and 1.8 mL of Hex [12]. These solutions were passed through preconditioned silica cartridges (3 mL, 500 mg, Waters, Wexford, Ireland) using 4 mL of ultrapure Milli-Q water saturated with EAc and 4 mL of Hex/EAc (90:10, *v*/*v*). Finally, these cartridges were rinsed with 3 mL of Hex/EAc (90:10, *v*/*v*), and the target compounds were eluted with 3 mL of Hex/EAc (38:62, *v*/*v*) [12]. The eluate was then dried and reconstituted in 0.5 mL of MeOH acidified with formic acid (0.10%, *v*/*v*). In summary, this protocol concentrated the analyzed PGs 4000-fold.

During this process, the recoveries of the evaluated PGs were analyzed by spiking matrix blank samples with the target compounds using concentrations 5 and 10 times higher than the method quantification limits (MQLs) for each target PG in triplicate during three different days [12]. The Supplement Material refers to the data concerning this step (Table S1).

### 2.4. LC-MS/MS Analysis

#### 2.4.1. LC-MS/MS System Requirements

The LC-MS/MS system was a Liquid Chromatograph Thermo Finnigan Surveyor HPLC System coupled to an LCQ Fleet™ Ion Trap Mass Spectrometer (Thermo Scientific, Waltham, MA, USA). The program used for data acquisition and processing was Xcalibur^TM^ version 2.

The mass spectrometer tuning parameters were optimized by direct injection of ten PGs standard solutions (2 mg/L) prepared in MeOH acidified with formic acid (0.10%, *v*/*v*), following a previously established protocol [13].

Separation was performed using an Avantor^®^ ACE Excel column (50 mm × 2.1 mm i.d., 1.7 μm) maintained at 30 °C. The mobile phases are 0.1% formic acid in Milli-Q water and 0.1% formic acid in MeOH (A) [12]. The linear gradient used was adapted from the procedure described by [11]. The mobile phase began at 50% A, followed by a gradual increase to 80% over 5 min. After that, A was increased to 90% in another 5 min and ramped up to 100% in just 0.1 min, where it stayed for 1 min. The injection volume was 10 μL, and the flow rate was set at 0.35 mL/min.

The operating conditions for the MS were as follows: capillary voltage = 30 V, capillary temperature = 350 °C, sheath gas (N_2_) pressure = 50 psi, auxiliary gas (N_2_) pressure = 10 psi, source voltage = 7 kV, and tube lenses set to 100 V [13].

PGs were analyzed using positive ionization mode in Total Ion Current mode (TIC, 30–500 *m*/*z*) and Collision-Induced Dissociation mode (CID), focusing on specific PG transitions (data in Supplement, Table S2). The data collection and analysis were completed using Xcalibur™ version 2.

#### 2.4.2. Quality Assurance (QA) and Quality Control (QC)

The evaluation of the equipment performance was conducted using blanks at both the beginning and conclusion of the analysis, employing solvent controls consisting of MeOH acidified with 0.10% (*v*/*v*) formic acid and matrix-matched calibration curves (CCs) implemented for every set of 10 samples [13,14]. The CCs used six nominal concentration levels, ranging from 5 to 500 µg/L for all PGs and 100 µg/L for P-d_9_ (IS, deuterated surrogate).

Method detection and quantification limits (MDLs and MQLs) were defined as MDL = 3.3(α/S) and MQL = 10(α/S); α represents the standard deviation of the blank (*n* = 10), and S is the average slope of the regression lines for each target compound. MDL values for each target PG are in Table 1.

### 2.5. Theoretical Bioconcentration and Pharmacological Effects of PGs in Fish Plasma

Mathematical models serve as an additional resource to forecast the potential bioaccumulation of toxicants, namely drugs, in non-target organisms. The fish plasma model leverages the correlation between the internal concentrations in target organisms (mainly humans) and those in non-target organisms (e.g., fish) to estimate the probability of adverse effects based on the premise of the evolutionary conservation of drug targets.

Here, the bioconcentration factors in fish plasma (*BCF_FP_*) were calculated using Equation (1), a formula that has previously been employed to estimate this parameter in over 500 pharmaceutical compounds [15].

(1) $$\log{BCF}_{FP}=0.73\times logKow-0.88,$$

The hypothetical predicted effect (*PEC*) of the measured PGs in the plasma of fish, was calculated following the expression (2) [8,16]. Herein, *C_FP_* refers to the concentration of each PG in fish plasma.

(2) $$PEC=\frac{C_{FP}}{{BCF}_{FP}},$$

### 2.6. Preliminary Risk Assessments for the Environmental PGs

The possible ecotoxicological risk for aquatic organisms resulting from exposure to PGs (given the environmental levels found in this study) was calculated as shown in (3), based on the *RQs* [17,18]:

(3) $$RQ=\frac{MEC}{PNEC}$$

In the formula, *MEC* is the maximum “measured environmental concentration”, and *PNEC* refers to the “predicted no-effect concentration” for each substance. The *PNEC* values were derived as previously described [2], and the *RQ* scale was developed based on the findings of Wentzel et al. [19]. According to the latter, RQ values < 1.0 suggest no significant risk to local ecosystems. When the *RQ* falls between 1.0 ≤ *RQ* < 10, there is a minor potential for adverse effects. When the *RQ* falls between 10 ≤ *RQ* < 100, there is a substantial potential for adverse effects, and *RQ* ≥ 100 indicates that adverse effects ought to be anticipated [19].

### 2.7. Statistical Analysis

Descriptive and inferential statistics were calculated using PAST 4.02 [20] and GraphPad Prism (6.01, GraphPad Software, Inc., San Diego, CA, USA). The data presented in tables are expressed as a mean escorted by standard deviation (SD), whereas the graphical representations utilize boxplots (median, maximum, minimum, and first and third quartiles).

Values that fell below the MDLs were accepted in accordance with the guidelines established by the United States Environmental Protection Agency (EPA/240VB-06/003 2006), i.e., data = MDL/(√2).

The assumptions of normality and homogeneity of variance were verified using the Shapiro–Wilk W and Levene tests. Subsequently, a one-way analysis of variance (ANOVA) was conducted to compare independent sites and compound groups. Post-hoc comparisons were carried out using Tukey’s test. In instances where parametric assumptions were invalid and data transformation was ineffective, the non-parametric ANOVA (Kruskal–Wallis test) was utilized, followed by the Dunn’s post-hoc test. The significance threshold was set at α = 0.05.

PCA was performed using the correlation matrix, and the principal components (PCs) were derived based on the Kaiser criterion (eigenvalue > 1), the Scree Plot criterion, and the requirement of % variances exceeding 85% [21]. Additionally, linear discriminant analysis (LDA) was applied to enhance the differentiation between predefined groups, maximizing the variance between them while minimizing within-group variability [22].

## 3. Results

### 3.1. PGs Levels in the Douro River Estuary Surface Waters

Table 1 presents the average ± SD annual concentrations of ten PGs across ten sampling sites in surface waters (ng/L). The average sum of the concentrations of all PGs ∑[PGs] was 48.1 ± 2.5 ng/L. On average, the levels of ∑[Gonanes] were 15.0 ± 5.0 ng/L, whereas those derived from progesterone and spirolactone were 12.2 ± 6.2 ng/L and 4.2 ± 1.9 ng/L, respectively. The ∑[Natural PGs] concentrations were 9.4 ± 1.8 ng/L.

Table 1 also shows the MDLs values and the percentage of the detection rates for the current compounds. On average, those values and rates were 0.4 ng/L and 86.3%, respectively. Statistics referring to data in Table 1 revealed that the concentrations of ∑[Gonanes] were similar to those of ∑[Progesterone derivatives]. In contrast, the last groups of PGs revealed higher concentrations than those of DSP (*p* < 0.002) and ∑[Natural PGs] (*p* < 0.03). Furthermore, the concentrations of ∑[Natural PGs] were superior to those of DSP (*p* < 0.001).

**Figure 2:**
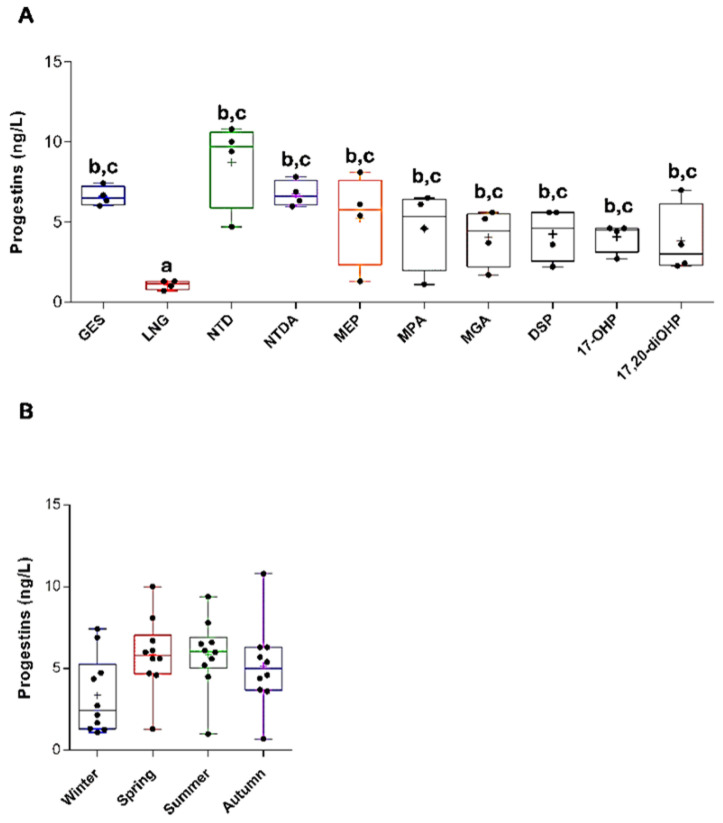
Concentrations (ng/L) by target PGs (**A**) and seasons (**B**). Data are expressed in boxplots with the minimum, median, maximum, average (+), and interquartile ranges Q1–Q3. In (**A**) dots indicate the average values recorded for each PG by season (*n* = 4), whereas those in (**B**) show the average levels of all PGs by season (*n* = 10). Significant differences in annual PG concentrations are indicated by different letters (**A**) (ANOVA, *p* < 0.05). No significant differences were detected among sampling sites or across seasons (**B**) (ANOVA, *p* > 0.05).

When comparing the individual compounds, ANOVA was significant, and the post-hoc analysis showed that LNG had lower concentrations than all other PGs (Figure 2A). However, no significant seasonal variations were found when considering the sum of all PGs in winter, spring, summer, and autumn (Figure 2B).

**Figure 3:**
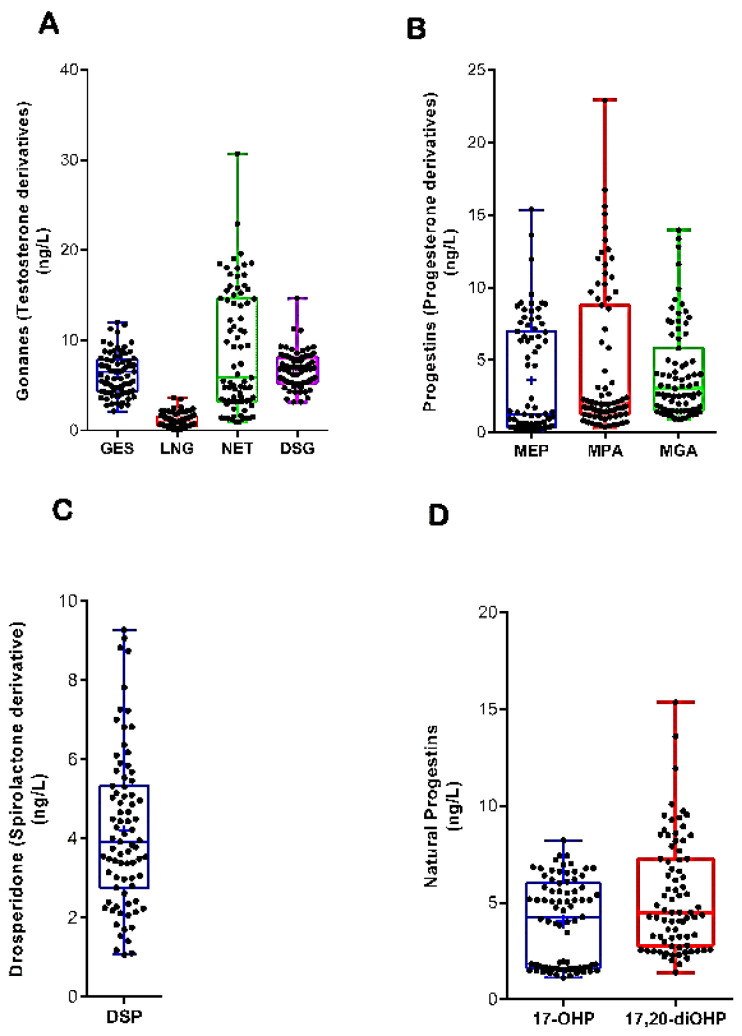
Levels (ng/L) of (**A**) gonanes, (**B**) progesterone derivatives, (**C**) drospirenone, and (**D**) natural PGs. Results are expressed in boxplots with the minimum, median, maximum, average (+), and interquartile range Q1–Q3. Dots represent individual values measured on different sampling occasions at each sampling site (*n* = 80). No significant differences were observed amongst PGs of the same group (ANOVA, *p* > 0.05).

No significant differences were also examined within each group of PGs. The overall concentrations of GES (5.0 ± 1.3 ng/L), LNG (2.3 ± 1.4 ng/L), NTD (8.7 ± 3.1 ng/L), and NTDA (6.3 ± 2.2 ng/L) are shown in Figure 3A. The levels of progesterone derivatives did not differ either; the average concentrations of MEP, MGA, and MPA were 3.6 ± 1.4 ng/L, 4.1 ± 2.0 ng/L, and 4.6 ± 3.0 ng/L, respectively (Figure 3B). Figure 3C,D show the annual amounts of DSP (4.2 ± 1.9 ng/L) and natural progestins. Relative to the latter, the concentrations of 17,20-diOHP (4.1 ± 0.8 ng/L) were like those of 17-OHP (5.3 ± 1.3 ng/L).

**Figure 4:**
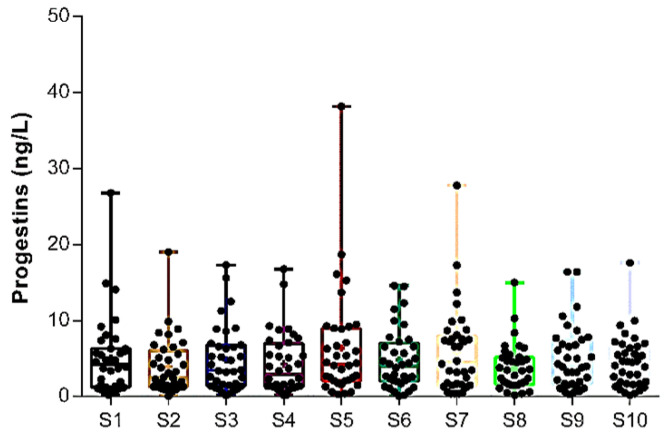
Global amounts of PGs in each Douro River Estuary sampling site. Results are expressed in boxplots with the minimum, median, maximum, average (+), and interquartile range Q1–Q3. Dots indicate the average individual values measured on different sampling occasions (*n* = 80). No significant differences were found among sampling sites (ANOVA, *p* > 0.05).

Figure 4 provides a comprehensive overview of the average annual concentrations of all synthetic PGs investigated by sampling site. No notable differences were found between the sites and margins of the Douro River Estuary.

**Figure 5:**
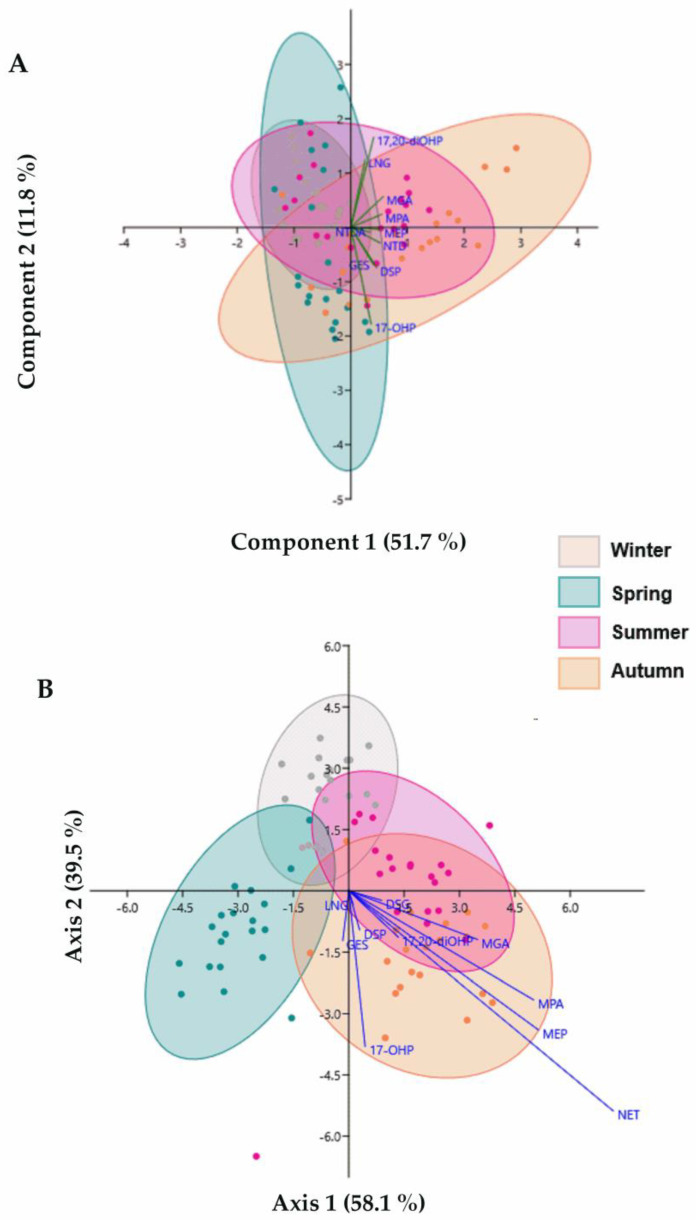
(**A**) Principal component analysis (PCA) score plots of PC1 vs PC2 show the distribution of individual PGs by sampling occasion (*n* = 4) and sites (S1 to S10, *n* = 10). (**B**) Linear discriminant analysis (LDA) score plots of Axis 1 vs。 Axis 2 illustrate the similarity between individual PGs by sampling season (*n* = 4) and sampling sites (S1 to S10, *n* = 10).

Figure 5A shows a PCA graphic (95% ellipses) considering PG content in each season and sampling site. When applying PCA to the current data, four principal components (PC1, PC2, PC3, and PC4) retained most of the variation (≈84%). MGA (0.40) is the principal contributor for PC1 and 17-OHP (−0.60) for PC2. For PC3 and PC4, the main contributors are GES (0.58) and LNG (0.54), respectively (Table S3). The PCA graphic shows that PGs’ variability is lower in winter and autumn, whereas spring and summer exhibit greater variability in the data.

In parallel, LDA (Figure 5B) maximized the ratio of the between-class variance. In this case, the LDA was employed to assess the differences linked to seasonal fluctuations of the 10 PGs in the 10 sampling sites. The model identified two functions that explained 97.6% of the variability (function 1–58.1%, function 2–39.5%). Thus, out of 800 parameters (10 PGs × (2 × 10 sampling sites) × 4 seasons), 20 were comprised in the model as being the best predictors and having the highest discriminating ability. For functions 1 and 2, the variables with the highest correlations were MEP (0.56) and NTDA (0.46). Figure 5B also shows a higher correlation of data obtained in winter, followed by autumn, spring, and summer (Table S4).

### 3.2. RQs for Environmental Synthetic PGs

Table 2 shows the prospective risk posed by PGs in surface waters. These data reveal that all assayed synthetic PGs except MEP, due to the absence of PNEC values, showed *RQs* ≥ 1. The top PGs with higher RQ values were GES, LNG, and NTD.

### 3.3. Predicted Effect of Environmental Concentrations of PGs

Table 3 shows the prospective amounts of the current PGs in fish plasma. The data suggest that NTD, NTDA, and MPA are likely in surface waters at concentrations equivalent to the therapeutic dosage ranges used by humans consuming these PGs for health purposes [8,23].

## 4. Discussion

Natural and synthetic PGs are common surface water micropollutants that pose significant risks to the biota [2,5]. Being excreted by humans and animals, PGs reach the environment through different sources—such as direct sewage, WWTPs effluents, hospitals, livestock farms, and aquacultures—in amounts that can surpass those of estrogens up to 1000-fold [24,25,26]. Moreover, it is well established that progesterone can be metabolized into over 20 products in various organisms, with hydroxylated metabolites being greatly more active in fish compared to the parental compound [27]. Furthermore, fish’s reproductive physiology and behavior can be influenced by exposure to both 17-OHP and 17,20-diOHP, even at concentrations < 1 ng/L [28,29]. Synthetic PGs are widely used in Europe, with Portugal among the highest consumers, mainly through contraceptive pills [30].

Considering the above, this research is the first to document the occurrence of PGs in the estuarine waters of the Douro River, the second-longest river in the Iberian Peninsula, and one of the few investigations in Portugal regarding this issue. This work is expected to serve as a comparative baseline for future studies in the surveyed ecosystem and a reference for others working in the field elsewhere and those involved in implementing policies for the depollution and remediation of aquatic habitats. So far, only two other studies have documented the occurrence of PGs in Portuguese aquatic environments, and these were restricted to LNG, GES, DSP, and desogestrel [13,31].

Globally, the current findings indicate that the Douro River Estuary has levels of PGs comparable to those observed in other aquatic systems [2], with annual average concentrations of ~38.8 ng/L for the eight assayed synthetic PGs and ~9.4 ng/L for the natural ones. These levels warrant serious concern due to their possible detrimental impacts on aquatic life [2,8]. As reviewed by Rocha and Rocha [2], these environmental concentrations can affect reproductive behavior, alter endogenous hormone levels, and influence sex differentiation in fish. Also, that review evidenced that these levels fall within the range known to cause deformities in the embryos and larvae of various aquatic species and disrupt swimming patterns, feeding behavior, and predator–prey interactions, promoting long-term impacts on animals’ growth rates and development.

Considering the testosterone-derived PGs, the average concentrations of GES in this study are ~10 times lower than those found in other studies conducted in Portugal [31]. However, they are significantly higher than levels reported in other European aquatic systems, such as those in Switzerland, where they are ~25 times higher [32], as well as in the Czech Republic (<0.05–<0.64 ng/L) and Germany (<0.3 ng/L) [3,33,34].

On average, the concentrations of LNG in the Douro River Estuary are roughly 5 times lower than those measured in other Portuguese estuarine surface waters [13]. In comparison to other European contexts, the LNG levels of this study are like those found in Hungary [35] but higher than those measured in Germany, where this PG was <MDL [7]. A comparable situation has been observed in Australia [36] and Canada [37,38]. Compared to Asia, the LNG levels found in the Douro River Estuary are about two times lower than those documented in Malaysia surface waters [12,39]. Globally, in this estuary, LNG is the PG with the lowest average concentrations. This is quite logical, considering that LNG is primarily used in emergency contraception pills, whereas other PGs are used in daily contraceptive pills and, as a result, are expected to appear in higher concentrations.

In analyzing NTD, its concentrations in the Douro River Estuary fall within the range observed in surface waters from Malaysia [39], China [40], and Canada [37]. As to NTDA, there is a scarcity of data to compare this study’s levels with those worldwide. Nevertheless, the concentrations appear to exceed those recorded in Germany, where the amounts were reported to be less than 0.3 ng/L [7].

In summary, the levels of gonanes found in the surface waters of the Douro River Estuary are, in general, higher than those reported in other urban-impacted aquatic environments around the world. We suggest that this occurrence can be a consequence of the passage of this river by important cities in both Spain (Valladolid, Salamanca, and Zamora) and Portugal (Porto and Vila Nova de Gaia), Portugal being one of the top countries where PGs are widely used [41] “https://www.epfweb.org/node/746 (accessed on 16 March 2025)”. Moreover, these data reveal some differences between this habitat and others, particularly those in southern Portugal, as evaluated by Morais et al. [13]. While the presence of these EDCs poses risks to local wildlife, it appears that the wastewater treatment plants (WWTPs) on both sides of the Douro River Estuary are more efficient at managing gonanes than those in other Portuguese aquatic habitats [13]. This is a plausible explanation because previous studies demonstrate that the percentages of removal by WWTPs of these PGs are very variable, ranging from negative values up to ~96% [2].

The PGs derived from progesterone (MEP, MPA, and MGA) have similar concentrations among each other (~5 ng/L). When these levels are compared to those reported globally, they align with the ranges found in aquatic systems in Brazil, China, Czech Republic, Germany, and Switzerland, i.e., MEP < 0.04–1.3 ng/L, MGA < 0.01–< 20.0 ng/L, and MPA < 0.01–0.31 ng/L [3,7,32,34,42,43]. Earlier research indicated that progesterone-derived PGs are typically found in surface waters at lower levels than gonanes [2]. Since this trend was not found in the Douro River Estuary, one probable reason for this difference is that Portugal ranks among the top countries in the use of combined pills, with over 62% of women aged 15 to 49 choosing this method as their primary form of contraception [44].

The spironolactone derivative DSP was measured in the Douro River Estuary in amounts comparable to those in other European aquatic habitats [35], despite the fact that most of the studies relative to the presence of this PG worldwide reveal that its levels are usually below the MDLs of the used methodology [2]. In contrast, a previous study conducted in different Portuguese aquatic systems reported levels approximately 40 times higher than those observed here [13]. One reason that could explain these differences may be linked to the good performance of the WWTPs in the Douro River Estuary compared to others in other Portuguese regions [13], as hypothesized above for gonanes.

No significant differences were found between margins, sampling sites, and seasons of the Douro River Estuary. This consistency indicates uniformity in the environmental conditions across the various sites examined, suggesting that the factors affecting the distribution of the studied parameters remained relatively stable throughout the year. Considering these findings, PCA was performed to uncover possible seasonal differences. This analysis revealed that summer showed distinct variation patterns in the distribution of the PGs, suggesting that this season is shaped by unique environmental or human influences. This observation is probably associated with the increase in tourist activity in the area during the summer, a trend also noticed in our earlier research on other hormones found in the surface waters of the Douro River Estuary [11] and reported in studies conducted in other Portuguese habitats [13].

Additionally, a follow-up LDA was performed to gain deeper insights into the seasonal profiles of PGs. This strategy shows that winter exhibited the highest data homogeneity, which logically agrees with Oporto’s more consistent resident population during this season. Therefore, the LDA analysis supports the previous hypothesis that tourism-related human activities significantly influence PGs’ distribution patterns in this habitat.

Concerning the natural progestins, the concentrations of 17-OHP in the Douro River Estuary are close to those in China, i.e., ~6 ng/L [45] and, overall, fall within the ranges reported in other parts of the world, i.e., 0.07–10 ng/L, namely in China, France, Hong Kong, South Africa, and the USA [9,12,46,47,48,49,50]. Yet, the present findings are ~8 times higher than those reported in Argentina (0.5 ng/L) [51]. Although the levels of 17,20-diOHP are frequently overlooked, this oversight is concerning, given the critical role that the hormone plays in the reproductive processes of numerous fish species. For example, it is involved in sperm motility and egg development, vital for the breeding success of fish populations and, consequently, the health of aquatic ecosystems [52].

Given the critical impact of the assayed PGs on fish physiology [2,9,52], their *RQs* values were calculated. The results were concerning, as all measured *RQs* have values higher than 1. This threshold indicates significant ecological risk, affirming that those contaminants negatively influence fish physiology. Indeed, elevated RQ values signify that the concentrations of PGs in the environment are high enough to disrupt normal biological functions in fish. As reported above, this can lead to various adverse effects, reinforced recently by elegant in vivo and in vitro studies [53,54,55]. Such physiological stressors can have cascading effects throughout the food web, impacting fish populations and the predators that rely on them, including birds and mammals. The risks are even more worrying for the Douro River Estuary when it is known that PGs are not the only organic and EDCs pollutants there [11,55,56,57,58].

Besides the risks backed by *RQs*, the theoretical provision of PGs’ concentrations in fish plasma, using the current MEC environmental concentrations, revealed that those of NTD, NTDA, and MPA are above or within the range of the therapeutic doses observed in humans. This finding raises significant concerns regarding the potential disruptive impact of the found PGs concentrations on the local aquatic life, particularly in fish. These concerns are further supported by the circumstance that many cell receptors and enzyme systems are preserved through both mammalian and non-mammalian species, which allows for the extrapolation of action mechanisms for specific compounds based on their environmental concentrations [8,59].

## 5. Conclusions

This study first investigated the presence, distribution, sources, and potential risks of ten natural and synthetic PGs in surface waters from the Douro River Estuary, exposing their ubiquitous occurrence in this habitat. No striking seasonal variations were noticed. However, higher data variability and a different profile on PGs were found in summer, backing up the hypothesis of a seasonal touristic influence. Also, the usually neglected natural progesterone metabolites (17-OHP and 17,20-diOHP) were found in significant concentrations. This is a step forward compared to previous studies concentrating only on the presence of synthetic compounds. A risk assessment indicated that the detected PG levels pose significant risks to aquatic organisms. NTD, NTDA, and MPA contribute the most to the total progestogenic activity, mainly because they should reach fish plasma amounts compatible with the therapeutic dosages reported for humans. The results should help policymakers conduct pollution control and risk management of PGs within the Douro River Estuary and highlight the need for monitoring and actions to manage and protect this ecosystem in the face of increasing anthropogenic pressures.

## Figures and Tables

**Table 1:** Annual concentrations of the ten PGs (mean, ng/L ± standard deviation, SD) recorded in the Douro River Estuary throughout 2023 (*n* = 80, comprising 10 sampling sites with independent duplicates collected in winter, spring, summer, and autumn). The method detection limits (MDL) are specified for each PG, along with their corresponding environmental detection rates (% DR), which indicate the frequency of occurrence in the analyzed samples.

PGs	MDL	DR (%)	S_1_	S_2_	S_3_	S_4_	S_5_	S_6_	S_7_	S_8_	S_9_	S_10_
	(ng/L)											
GES	0.3	79	5.6 ± 1.4	3.4 ± 0.7	3.6 ± 1.9	5.9 ± 3.1	6.3 ± 3.2	6.2 ± 3.4	5.7 ± 3.6	6.0 ± 3.7	4.3 ± 2.9	2.9 ± 1.7
LNG	0.3	81	5.3 ± 0.6	3.3 ± 3.2	1.4 ± 1.2	2.4 ± 2.1	2.7 ± 2.5	2.5 ± 2.5	1.3 ± 1.0	3.0 ± 3.5	0.6 ± 0.6	0.8 ± 0.5
NTD	0.7	90	4.4 ± 0.2	5.8 ± 3.9	5.5 ± 5.8	9.8 ± 5.3	11.3 ± 6.7	10.8 ± 6.2	9.8 ± 7.5	14.3 ± 10.6	9.4 ± 8.6	6.2 ± 4.5
NTDA	0.2	80	0.6 ± 0.4	5.2 ± 1.2	7.0 ± 1.5	7.3 ± 0.7	6.7 ± 1.7	8.0 ± 1.7	7.5 ± 1.1	8.4 ± 3.0	6.9 ± 1.6	5.0 ± 0.7
MEP	0.7	78	0.7 ± 0.7	2.4 ± 3.3	2.5 ± 3.8	5.6 ± 6.3	4.1 ± 4.0	4.1 ± 3.9	4.6 ± 4.5	4.5 ± 4.7	3.6 ± 3.7	3.5 ± 4.0
MGA	0.6	80	1.4 ± 0.1	1.9 ± 0.4	2.5 ± 1.0	7.6 ± 5.0	4.9 ± 2.9	5.3 ± 3.3	6.5 ± 4.8	4.3 ± 2.5	3.6 ± 2.3	2.6 ± 1.2
MPA	0.3	89	1.0 ± 0.3	1.2 ± 0.7	1.6 ± 0.8	8.1 ± 5.7	6.2 ± 5.4	8.3 ± 7.5	7.2 ± 5.7	4.9 ± 4.2	5.8 ± 5.3	1.5 ± 0.8
DSP	0.6	98	1.6 ± 0.4	2.6 ± 0.6	2.7 ± 0.7	7.6 ± 1.6	5.0 ± 0.8	5.6 ± 0.9	6.2 ± 1.4	4.3 ± 1.0	3.6 ± 0.7	3.3 ± 0.5
17-OHP	0.5	96	2.7 ± 2.5	3.2 ± 3.0	2.9 ± 2.3	4.9 ± 2.7	4.5 ± 2.4	4.8 ± 2.3	4.9 ± 2.3	4.4 ± 2.0	4.3 ± 1.9	3.9 ± 1.8
17,20-diOHP	0.2	92	3.8 ± 2.2	5.2 ± 2.9	5.7 ± 2.8	8.3 ± 4.8	4.7 ± 2.3	5.5 ± 2.9	6.0 ± 3.3	5.1 ± 2.7	4.1 ± 1.8	5.1 ± 0.7
∑Progestins by site			27.1 ± 1.9	34.2 ± 1.5	35.4 ± 1.9	67.5 ± 2.1	56.4 ± 2.3	61.1 ± 2.4	59.6 ± 2.2	59.2 ± 3.3	46.0 ± 2.3	34.8 ± 1.6
Global amounts of Progestins in Douro River Estuary							48.1 ± 2.5					

**Table 2:** Bioconcentration factor in fish plasma (*BCF_FP_*), concentration in the plasma of a fish (*C_FP_*), which relates to the human plasma therapeutical levels, and predicted effect concentration (PEC*w*) values. Data in bold correspond to those measured average concentrations (MEC) of PGs measured in Douro River surface waters that show values like or above PEC*w*.

PGs	Log Kow	*BCF_FP_* (ng/mL)	*C_FP_* (ng/L)	PEC*w* (ng/L)	MEC (ng/L)
GES	3.3	32	1.0	31	5.3
LNG	3.5	46	2.4	52	14.3
NTD	3.0	19	9.8	6.7	**6.3**
NTDA	4.0	108	9.8	2.7	**8.4**
MEP	3.5	47	1.0	21.0	5.6
MGA	4.0	110	-	<10	7.6
MPA	4.1	128	1.0	8.0	**8.3**
DSP	4.0	113	30.8	273	7.6

**Table 3:** Risk quotients (*RQs*) of eight synthetic PGs referred to in this study, applying the maximum average concentrations (MEC) of PGs determined in Douro River surface waters. *RQ* values were not calculated for MEP due to the lack of endpoint values for fish. All PGs show *RQs* > 1.

PGs	Endpoint Value (ng/L)	PNEC (ng/L)	MEC (ng/L)	*RQ*
GES	EC_50_ = 10	0.01	6.3	630
	AF = 1000			
LNG	NOEC = 0.42	0.01	5.3	530
	AF = 50			
NTD	LOEC = 4	0.08	14.3	179
	AF = 50			
NTDA	NOEC = 816	0.80	8.4	11
	AF = 1000			
MEP	-	-	5.6	-
MGA	NOEC = 33	0.66	7.6	12
	AF = 50			
MPA	NOEC = 342	6.84	8.3	1.2
	AF = 50			
DSP	NOEC = 100	2.00	7.6	3.8
	AF = 50			

## Data Availability

Data are obtainable from the corresponding author upon reasonable request.

## Supplementary Materials

The following supporting information can be downloaded at: www.mdpi.com/article/10.3390/toxics13030225/s1, Table S1: Retention times, quantification and diagnostic ions, of each target compound analyzed by LC-MS/MS; Table S2: SPE recoveries for two concentration levels (5 MQL and 10 MQL) of each target compound analyzed by LC-MS/MS. Recoveries are given as mean ± standard deviation (SD); Table S3: (A) Data referring to PCA of PGs and (B) to LDA in surface waters collected from the Douro River Estuary (Portugal).
