# Supplementary material for: Progestin Pollution in Surface Waters of a Major Southwestern European Estuary: The Douro River Estuary (Iberian Peninsula)
Source: Toxics. 2025 Mar 19;13(3):225. doi: 10.3390/toxics13030225 (PMC11946473; doi:10.3390/toxics13030225)
Supplement: Supplementary file 1 [file toxics-13-00225-s001.zip › Table S1 - LC-MSMS parameters.pdf]

Table S1

Target compounds	Retention time (min)	Base Peak $[M + H]^+$ (m/z)		Confirming fragments (m/z)	CID (eV)
Norethindrone (NTD)	4.7	299	→	145, 231	25
Drospirenone (DSP)	5.5	367	→	131,159	30
17α-hydroxyprogesterone (17-OHP)	5.6	331	→	97, 313	30
17α,20β-dihydroxy-4-pregnen-3-one (17,20-diOHP)	6.0	333	→	97,271	30
Levonorgestrel (LNG)	6.3	313	→	187, 245	20
Desogestrel (DSG)	6.6	293	→	265,197	30
Medroxyprogesterone (MEP)	6.9	345	→	267, 327	30
Megestrol acetate (MGA)	7.4	385	→	224, 267	30
Progesterone-d_9 (P-d_9)	7.5	324	→	100, 113	30
Norethindrone acetate (NTDA)	7.5	341	→	143, 299	30
Medroxyprogesterone acetate (MPA)	7.6	387	→	123, 285	30
