# Supplementary material for: Progestin Pollution in Surface Waters of a Major Southwestern European Estuary: The Douro River Estuary (Iberian Peninsula)
Source: Toxics. 2025 Mar 19;13(3):225. doi: 10.3390/toxics13030225 (PMC11946473; doi:10.3390/toxics13030225)
Supplement: Supplementary file 1 [file toxics-13-00225-s001.zip › Table S2 - SPE recoveries.pdf]

Table S2

Target compound	5 x MQL (ug/L)	10 x MQL (ug/L)	Recoveries (% ± SD)					
			5 x MLQ			10 x MLQ		
Gestodene (GES)	17.0	34.0	89.6	±	1.3	95.7	±	1.1
Levonorgestrel (LNG)	21.0	42.0	77.7	±	1.0	93.7	±	3.1
Norethindrone (NTD)	48.5	97.0	79.6	±	0.7	87.0	±	6.1
Norethindrone acetate (NTDA)	11.5	23.0	80.0	±	0.9	96.2	±	1.0
Medroxyprogesterone (MEP)	46.0	92.0	87.3	±	2.3	97.5	±	4.0
Megestrol acetate (MGA)	22.5	45.0	85.3	±	1.5	95.3	±	2.3
Medroxyprogesterone 17-acetate (MPA)	39.0	78.0	88.7	±	0.6	95.9	±	6.6
Drospirenone (DSP)	43.0	86.0	82.3	±	1.7	95.0	±	3.5
17α-hydroxyprogesterone (17-OHP)	33.5	67.0	81.3	±	0.3	96.0	±	3.8
17α,20β-dihydroxy-4-pregnen-3-one (17,20-diOHP)	15.0	30.0	88.7	±	0.2	98.3	±	0.2
