# Supplementary material for: Progestin Pollution in Surface Waters of a Major Southwestern European Estuary: The Douro River Estuary (Iberian Peninsula)
Source: Toxics. 2025 Mar 19;13(3):225. doi: 10.3390/toxics13030225 (PMC11946473; doi:10.3390/toxics13030225)
Supplement: Supplementary file 1 [file toxics-13-00225-s001.zip › Table S3.pdf]

Table S3

PCA	Eigenvalue	% variance
1.0	5.17	51.71
2.0	1.18	11.76
3.0	1.09	10.90
4.0	0.92	9.15
5.0	0.62	6.23
6.0	0.35	3.48
7.0	0.32	3.15
8.0	0.17	1.74
9.0	0.13	1.28
10.0	0.06	0.60

	PC 1	PC 2	PC 3	PC 4	PC 5	PC 6	PC 7	PC 8	PC 9	PC 10
**GES**	0.28	-0.23	**0.58**	-0.04	-0.01	-0.62	0.29	-0.22	0.15	0.01
**LNG**	0.18	0.42	0.41	**0.54**	0.52	0.14	-0.17	0.14	0.04	0.00
**NTD**	0.36	-0.09	-0.34	-0.01	0.39	-0.24	0.06	-0.08	-0.72	0.00
**NTDA**	0.24	-0.03	0.16	-0.73	0.45	0.33	0.07	0.15	0.19	0.00
**17-OHP**	0.25	**-0.60**	-0.13	0.39	0.05	0.31	0.37	0.26	0.19	-0.27
**17,20-diOHP**	0.28	0.55	-0.04	-0.01	-0.32	0.19	0.67	0.00	-0.09	0.08
**MEP**	0.38	-0.01	-0.38	0.12	0.10	0.04	-0.12	-0.51	0.45	0.46
**MPA**	0.39	0.08	-0.16	-0.04	-0.22	-0.34	-0.25	0.72	0.15	0.22
**MGA**	**0.40**	0.19	-0.07	-0.07	-0.21	0.00	-0.33	-0.23	0.10	-0.76
**DSP**	0.32	-0.25	0.40	0.00	-0.40	0.42	-0.31	-0.10	-0.37	0.29
