# Supplementary material for: Progestin Pollution in Surface Waters of a Major Southwestern European Estuary: The Douro River Estuary (Iberian Peninsula)
Source: Toxics. 2025 Mar 19;13(3):225. doi: 10.3390/toxics13030225 (PMC11946473; doi:10.3390/toxics13030225)
Supplement: Supplementary file 1 [file toxics-13-00225-s001.zip › Table S4.pdf]

Table S4

Eigenvalue	%
4.3	58.1
3.2	39.5
0.4	5.6
0.2	2.2

	**Axis 1**	**Axis 2**	Axis 3	Axis 4
**GES**	-0.20	-0.05	-0.30	-0.13
**LNG**	-0.05	-0.49	0.11	-0.09
**NTD**	0.00	0.08	0.01	-0.12
**NTDA**	0.03	-0.14	0.37	0.14
**17-OHP**	-0.42	-1.08	0.12	0.53
**17,20-diOHP**	-0.35	-0.24	-0.01	-0.14
**MEP**	**0.56**	0.00	-0.04	-0.41
**MPA**	0.06	0.02	-0.31	0.02
**MGA**	0.33	0.07	0.34	0.82
**DSP**	-0.25	**0.46**	0.31	-0.76
